# Spatial and temporal variations in female size at maturity of a Southern Rock Lobster (*Jasus edwardsii*) population: A likely response to climate change

**DOI:** 10.1371/journal.pone.0225144

**Published:** 2019-11-11

**Authors:** Lachlan J. McLeay, Mark J. Doubell, Adrian J. Linnane

**Affiliations:** South Australian Research and Development Institute (Aquatic Sciences), Adelaide, South Australia, Australia; Fisheries and Oceans Canada, CANADA

## Abstract

The size at which sexual maturity is reached is a key population parameter used to guide the setting of minimum legal size limits in fisheries. Understanding spatial and temporal variations in size at maturity is fundamental to management because the relationship between size at maturity and minimum legal size limits affects the fraction of the mature population biomass that is harvested, and resulting egg production, larval settlement and recruitment. This study measured the size at maturity of female Southern Rock Lobster (*Jasus edwardsii*) across South Australia between 1991 and 2015 in relation to known oceanographic characteristics, surface and subsurface temperature data, and relative changes in lobster abundance. There was pronounced north to south spatial variation in estimates of size at maturity. Larger average size at maturity was recorded in warmer north-western areas of the fishery relative to the cooler waters of the south-east. Estimates of size at maturity also differed over 25 years across the fishery. However, the nature of temporal responses varied spatially, and were more consistent with variations in surface and subsurface water temperature at local-scales than changes in lobster density. In the well-mixed waters of the north-western, western and south-eastern parts of the fishery, relatively high rates of increase in sea-surface temperature and size at maturity were recorded since 1991, indicating that size at maturity may be responding to ocean warming associated with global climate change. In more central parts of the fishery, contrasting temporal signals in sea-surface temperature (positive) and bottom temperature (negative) indicated increases in upwelling strength over the study period, and formation of a bottom cold pool below a warm surface layer, with corresponding decreases in size at maturity recorded. The spatio-temporal changes in size at maturity measured in this study highlight the need for oceanographic information to be integrated into future stock assessment models to enhance harvest strategy development, allow timely adaptive management decisions and increase the resilience of fisheries to the impacts of climate change.

## Introduction

Fisheries management requires stock assessments to account for variations in the life history traits of fish populations that alter the way they respond to fishing pressure [1][2]. The size at onset of sexual maturity, herein referred to as size at maturity, is a key population parameter used to guide the setting of minimum legal size limits in fisheries worldwide, yet may vary spatially and temporally in response to factors such as age, growth rate, temperature, population density, food availability or their interaction [3][4][5]. In lobster fisheries, minimum legal size limits are routinely set using the average length (carapace length—CL) at which 50% of females are mature (L_50_), based on the assumption that this affords juvenile stages protection from fishing mortality and allows at least 50% of females to spawn at least once before being harvested [6]. While minimum legal size limits are often set, understanding spatial and temporal variations in size at maturity across a fished stock is crucial to fisheries management because the relationship between minimum legal size limits and size at maturity is intrinsically linked to the fraction of mature biomass that is harvested, and resulting egg production, larval settlement and recruitment [7][8].

**Fig 1:**
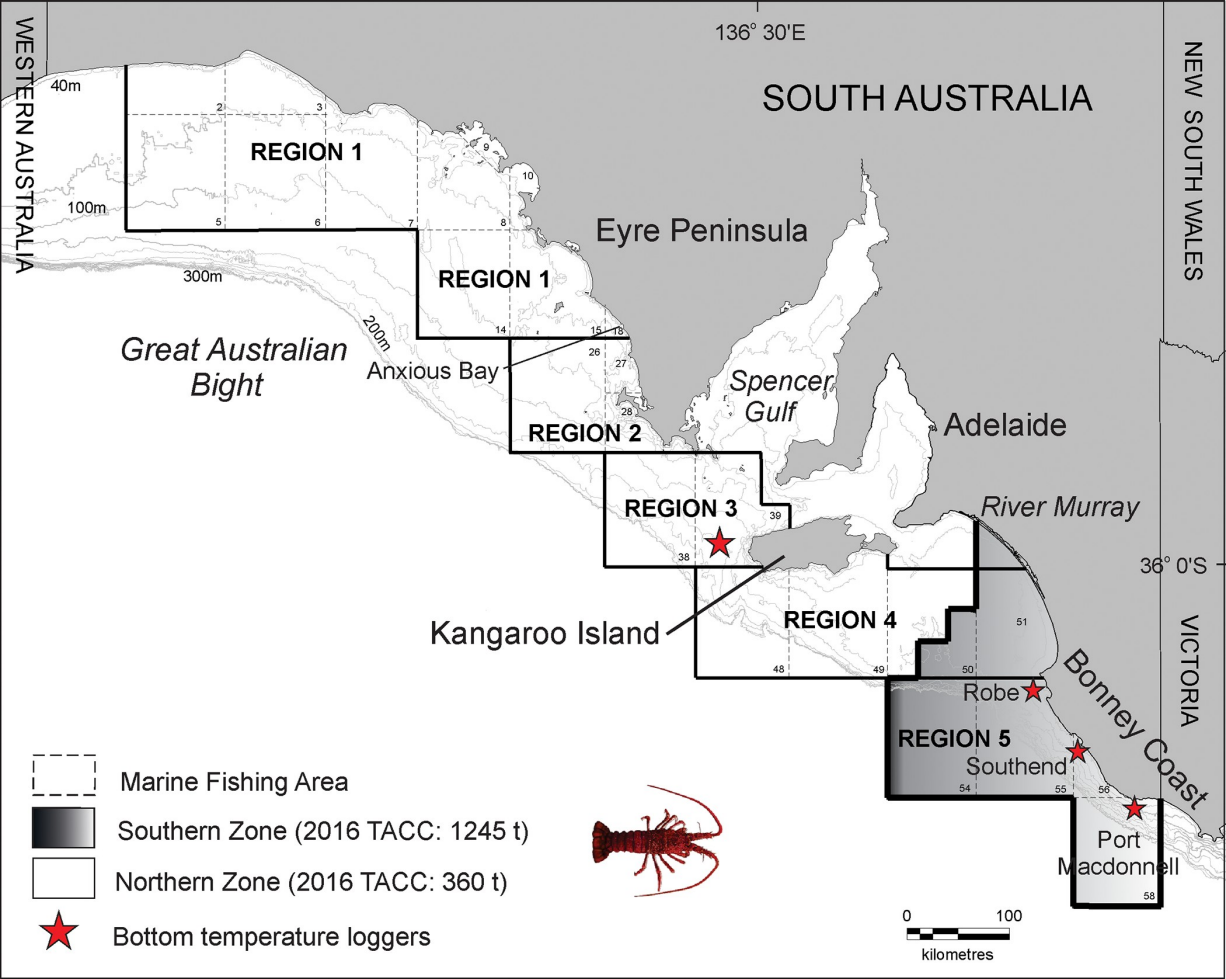
Map showing: The location of regions 1 to 5 used to analyse temporal patterns in sea-surface temperature, relative abundance (CPUE) and size at maturity (L_50_); Marine Fishing Areas and Management Zones used for management of the South Australian Rock Lobster Fishery; and the location of bottom temperature loggers. Region 4 is a combination of both the Northern and Southern Zone management units.

Southern Rock Lobster (*Jasus edwardsii*) (Hutton 1875) are a key target of regional fisheries of Australia’s southern continental shelf between Geraldton in Western Australia (29° S, 114° E) and Coffs Harbour in northern New South Wales (30° S, 172° E) [9], and have an annual commercial catch of ~3000 t valued at ~AUD $250 million in 2016/17 [10][11]. In South Australia, Southern Rock Lobster are the state’s most valuable fisheries resource, with an annual landed value of AUD $120 million in 2016/17 [11]. Fishing by the South Australian Rock Lobster Fishery is undertaken using baited traps within two management areas, the Northern Zone and Southern Zone (Fig 1). The division into two zones reflects ecological and biological characteristics that differ in both regions in relation to habitat [12], growth [13] and recruitment [14]. Fishing in both zones is managed using a suite of controls including limited entry, vessel-size and power restrictions, protection of ovigerous females, and seasonal and spatial closures. In addition, a Total Allowable Commercial Catch (TACC) has been in place in the Southern Zone since 1993 and in the Northern Zone since 2003 [15][16] (Fig 1). Individual minimum legal size limits that reflect spatial differences in growth [13] and size at maturity [17][18] have been set in each zone to protect brood stock and maintain egg production. In the Southern Zone, the minimum legal size limit of 98.5 mm carapace length (CL) has been in place since 1970 [16]. In the Northern Zone, minimum legal size limits have changed over time, increasing from 98.5 to 102 mm CL in 1995, and further to 105 mm CL in 2000 [15].

Recruitment of Southern Rock Lobster has declined across the species’ distribution in southern Australia, resulting in declines in fishery performance in all jurisdictions where lobster are targeted [15][16][19][20]. Total catches in each state are currently some of the lowest on record, and recent assessments indicate that combined levels of egg production are approaching the limit reference point of 20% unfished egg production used in national assessment of the stock [21]. The broad spatial-scale over which the declines in recruitment have been recorded signal the occurrence of a regime shift associated with a change in environmental conditions, rather than the effects of fishing-related mortality alone [22]. In Tasmania, hind-cast stock assessment modelling identified a negative relationship between larval settlement, model-estimated recruitment and sea-surface temperature [23]. However, the study noted considerable variation in the relationship between different regions of the fishery, and highlighted the need for studies to examine biophysical relationships at spatial scales relevant to existing local oceanographic processes.

Crustacean sensitivity to changes in their local thermal environment is well recognised (reviewed in [24]), yet the impacts of increasing ocean temperatures associated with climate change on populations traits are not yet well documented. For lobsters, increases in water temperature can accelerate growth and development, yet the nature of the relationship between size at maturity and temperature is not consistent among species [25][26][27][28]. For example, in Western Rock Lobster (*Panulirus cygnus*) and American Lobster (*Homarus americanus*), size at maturity is inversely related to the temperature conditions experienced during growth [3][6][29][30]. Conversely, for Southern Rock Lobster, size at maturity is positively correlated with water temperature or proxies thereof (e.g. latitude, depth) [17][31][32][33]. Such divergent responses among species are thought to reflect species-specific adaptations in physiology that mediate the way temperature influences the partitioning of energy resources to processes of somatic growth and reproductive development [34]. Increases in temperature can either act to accelerate the intermoult period (moult frequency), increase the amount of growth per moult (moult increment), or both [24]. Consequently, where size at maturity is dependent on the number of moults (instars) before maturity is reached, reductions in size at maturity may be observed in response to higher temperature. Where size at maturity is independent of moult frequency, increases in size at maturity may occur in response to higher temperatures. In Southern Rock Lobster and other *Jasus* species, size at maturity is considered to be age dependent [32][35] and therefore independent of moult frequency. As a result, increases in water temperature lead to larger size at maturity as growth rates increase [17][31][32][33].

The intrinsic link between temperature, growth, size at maturity, and resulting egg production and recruitment in crustacean populations highlights the need for fishery resource managers to consider how fishery population traits might respond to changes in environmental conditions. This study uses data provided through long-term stock assessment monitoring in the South Australian Rock Lobster Fishery to measure temporal and spatial patterns of size at maturity of Southern Rock Lobster since 1991. We examine spatial and temporal patterns in size at maturity in relation to the known oceanographic characteristics in each part of the fishery, as well as surface and subsurface temperature data collected from satellites and ocean sensors. As density-dependent processes can also influence patterns in size at maturity, we also assess the effect of any relative changes in Southern Rock Lobster abundance on the patterns in size at maturity observed. Implications for stock assessment and management of the South Australian Rock Lobster Fishery are considered in relation to the patterns in size at maturity across South Australia and the current minimum legal size limits used to manage the fishery.

## Methods

### Study area and local oceanography

The study area encompasses waters targeted for fishing of Southern Rock Lobster by the South Australian Rock Lobster Fishery. The fishery is split into two management zones, the Southern Zone and Northern Zone, and each zone is subdivided into Marine Fishing Areas for reporting and management purposes (Fig 1). To analyse spatial and temporal trends in size at maturity, and the factors potentially influencing size at maturity, all data were assigned to 1 of 5 spatial regions (Fig 1). The spatial boundaries of the five regions were chosen to capture differences in the oceanographic features across the study area, including meridional changes, which influence sea-surface temperature [36] and variations in the location of upwelling centres [37]. The sizes of regions chosen also ensured a sufficient sample size of female lobsters in each year were available to undertake size at maturity analyses.

Local oceanographic conditions across the study area reflect differences in the nature of interactions between bathymetry and seasonal changes in atmospheric forcing [38]. During summer, prevailing south-easterly winds across the study area cause large-scale coastal upwelling events that vary in their intensity and duration depending on the orientation and bathymetric characteristics of the continental shelf [39][40]. The relatively narrow shelf and steep bathymetry at the southern tip of Eyre Peninsula in the eastern Great Australian Bight and off south-western Kangaroo Island (region 3), and the Bonney Coast (region 5), supports two to four upwelling events per summer, each occurring for over one week’s duration [37][38], with surface SST responses most evident on the Bonney Coast (Fig 1). These events pull cold water (<15°C) onto the shelf within 10km of the coast [40]. In region 3, cooler (<15°C), nutrient rich waters are upwelled from depths below 200m onto the shelf and reside in the bottom layer referred to as the Kangaroo Island cold pool [40][41]. The influence of upwelling diminishes to the west of region 3 across the Great Australian Bight, with upwelling events becoming shorter and restricted to near-shore areas of the coast as far west as Anxious Bay (region 1) [40] (Fig 1).

As the continental shelf broadens across the central Great Australian Bight (regions 1 and 2), downwelling at the shelf break [42] is expected year round [38][43], and summer warming of shallower shelf waters results in the establishment of a deep warm isothermal layer, known as the Great Australian Bight warm pool, with near uniform temperatures >17°C observed to depths greater than 40 m [40] (Fig 1). Similarly, Region 4 is characterised by a broad and shallow shelf area. Here, inshore waters are characterised by reduced wave energy and river discharge from Australia’s largest river system, the River Murray, while offshore waters are downstream from Bonney Coast upwelling events. These summer differences in oceanography between regions 1–5 are thought to influence the ontogenetic characteristics of Southern Rock Lobster populations in different parts of the South Australian fishery [19]. In contrast, winter oceanographic processes across South Australia are characterised by an increase in the intensity of Leeuwin current that transports warmer water from Western Australia east along the continental shelf, with connectivity extending to the east of Kangaroo Island in July [44]. Seasonal cooling and prevailing westerly winds, also drive coastal currents eastward [45] and drive deep downwelling to depths of 200m across the study region [46][47].

### Data sources

Data in the South Australian Rock Lobster Fishery are collected in each management zone through two data collection programs; 1) a commercial logbook program; and 2) a voluntary catch sampling program. Mandatory reporting of data through the commercial logbook program was implemented in 1970 as a requirement of fishing licence conditions. The logbook records data relating to fishing activities, including Marine Fishing Area, weight of Southern Rock Lobster catch, number of legal-size Southern Rock Lobster landed, and fishing effort as potlifts. The voluntary catch sampling program was implemented in 1991. Fishers and on-board observers voluntarily record information relating to the size (carapace length (mm, CL), sex, sexual maturity of female lobsters, as well as the date, depth and location of capture. Female lobsters are classified as sexually mature based on the presence of eggs or existence of ovigerous (long) setae on the endopodite of the pleopods [48]. Lobsters with ‘short’ setae are classified as immature. Between 1991 and 2015, fishers and on-board observers participating in the voluntary catch sampling program recorded data from a total of 729,567 lobsters. In this study, all fishery data are collected from a fishing season that occurs between October and May and we refer to year as the year in which each fishing season began (e.g. October 1999 to May 2000 = 1999). No permits are required to record data from fish or crustacean species targeted and landed by commercial fishing operations in South Australia.

Marine temperature data were derived from two sources. To assess temporal trends in sea-surface temperature, available data were obtained from the Integrated Marine Observing System (IMOS) portal [49] for each fishing season period (1 October to 31 May) between 1992 and 2015. Data downloaded from IMOS are obtained from an Advanced Very High Resolution Radiometer (AVHRR) instrument onboard the NOAA 19 polar-orbiting satellite. Temporal trends in bottom temperature data were obtained from loggers deployed in two regions of the study area (Fig 1). In region 3, bottom temperature data were collected from an IMOS mooring deployed at 100m off western Kangaroo Island between 2008 and 2016. The mooring forms part of the IMOS series of National Reference Stations (IMOS platform code: NRSAKAI) that are deployed to monitor ocean climate in Australian coastal ocean waters. Time-series measures of temperature at 100 m depth were collected using FSI/Teledyne Conductivity, Temperature, Depth (CTD) instruments each sampling for 15 s at 2 Hz. In region 5, bottom temperature data were collected from onset® StowAway®TidbiT® TBI32-05+37 bottom loggers, with an accuracy of ±0.2° at 20°C and a response time of 60 min, deployed at 60m depth at Southend, Robe and Port MacDonnell between 1998 and 2016 (Fig 1).

### Data analysis

Previous research in the Southern Zone of the South Australian Rock Lobster Fishery identified a negative relationship between size at maturity and depth [18]. Since 1993, fishing operations in the Southern Zone have also contracted inshore following the introduction of quota [50]. We considered that temporal analyses of size at maturity across the fishery could potentially be confounded by sampling biases associated with changes in water temperature linked to the movement of fishing operations inshore. To assess the need for our size at maturity analyses to account for temporal changes in fishing depth over time, we analysed data collected from the commercial logbook program since 1991 using linear regression in OriginPro®, with year as the dependent variable, to assess trends in the average depth of fishing in regions 1–5. To map the extent of any change in depth of fishing since 1991, we analysed the average percentage change in the depth of fishing operations in each Marine Fishing Area in two 8-year periods: 1991–1998 and 2008–2015.

A total of 372,109 female lobsters were measured, sexed and assessed for reproductive state in the voluntary catch sampling program between 1991 and 2015. Of these, 352,985 had sufficient information recorded to enable size at maturity analyses. Analyses of fishery data indicated movement of fishing operations to shallower inshore fishing grounds across all areas of the fishery since 1991 (see Results) so we undertook size at maturity estimation procedures in each region twice, once using the complete data set and again using data recorded from Southern Rock Lobster caught at 20–30m depth only (Table 1). Preliminary analyses indicated that this depth range was the most commonly fished in each region thereby providing the largest sample sizes for size at maturity analyses for each combination of region and year. Size at maturity was estimable for nearly all combinations of region (1–5) and year between 1991 and 2015. The exception to this was in region 1, where size at maturity was inestimable for some years (Table 1) due to low numbers of female Southern Rock Lobster sometimes measured in this region, which is indicative of the relatively low catch and observer effort in this part of the fishery.

**Table 1:** Number of female Southern Rock Lobster (*Jasus edwardsii*) measured by fishers and on-board observers from all depth contours and the 20-30m depth contour in fishery-dependent stock assessment surveys between 1991 and 2015. Region localities are shown in Fig 1. *indicates years low sample sizes precluded size at maturity (L_50_) estimation.

	ALL DEPTH CONTOURS						20–30m DEPTH CONTOUR					
	REGION						REGION					
Year	1	2	3	4	5	Total	1	2	3	4	5	Total
1991	881	2,797	3,115	5,273	9,741	21,807	258	534	750	570	2,763	4,875
1992	1,639	4,285	3,813	4,671	11,202	25,610	366	608	588	385	1,472	3,419
1993	126	1,398	1,593	1,876	7,442	12,435	9*	244	226	172	836	1,487
1994	189	1,145	496	873	1,813	4,516	26*	342	100	90	252	810
1995	919	1,693	963	1,625	6,942	12,142	217	296	134	210	1,350	2,207
1996	771	1,369	1,976	1,460	8,249	13,825	162	337	447	129	955	2,030
1997	814	1,363	2,082	2,095	9,270	15,624	301	270	424	282	1,181	2,458
1998	1,317	597	1,201	3,765	6,005	12,885	635	126	306	531	1,035	2,633
1999	784	619	1,029	1,813	10,190	14,435	195	115	248	270	1,623	2,451
2000	1,093	1,014	1,755	2,889	13,343	20,094	339	221	475	366	911	2,312
2001	1,209	814	848	2,429	9,966	15,266	338	195	218	363	2,506	3,620
2002	280	798	842	904	6,108	8,932	87	151	182	191	1,593	2,204
2003	195	731	893	1,584	10,344	13,747	31*	112	258	205	2,333	2,939
2004	399	660	1,328	1,682	12,128	16,197	84	213	237	161	2,577	3,272
2005	766	1,570	1,911	2,381	7,408	14,036	221	386	363	255	1,236	2,461
2006	238*	1,380	1,283	1,786	12,710	17,397	31*	370	363	316	3,635	4,715
2007	149*	756	415	1,357	10,373	13,050	61*	127	79	193	2,079	2,539
2008	221	664	876	1,231	8,316	11,308	52*	160	273	293	1,568	2,346
2009	266	2,860	2,106	1,298	8,714	15,244	39*	993	296	241	1,611	3,180
2010	76	672	1,268	1,611	8,927	12,554	18*	94	271	185	1,407	1,975
2011	550	1,189	1,729	965	8,171	12,604	253	334	515	89	1,723	2,914
2012	693	2,093	4,053	4,249	10,080	21,168	332	508	1137	540	2,079	4,596
2013	55	617	2,927	3,243	3,601	10,443	11*	131	702	899	855	2,598
2014	60*	2,159	1,419	1,199	3,696	8,533	24*	668	557	174	767	2,190
2015	83	941	1,624	1,309	5,176	9,133	34*	223	406	443	742	1,848
**Grand Total**	**13,773**	**34,184**	**41,545**	**53,568**	**209,915**	**352,985**	**4,124**	**7,758**	**9,555**	**7,553**	**39,089**	**68,079**

The size at maturity of female lobsters for each combination of region (1–5) and year between 1991 and 2015 was estimated using the SizeMat package (version 0.2.0) within R statistical software (v 3.2.2, R Development Core Team, R Foundation for Statistical Computing). To estimate size at maturity, a logistic regression of the form:

y=1/(1+exp−(a+b*x)) Eq 1

was first fitted to the proportion of mature females in each 5-mm CL class, where *y* is the probability of an individual being mature at determinate *x* (carapace length (mm)) and *a* (intercept) and *b* (slope) are estimated parameters. Size at maturity (*L*_*50*_) was then calculated as the average carapace length at which 50% of females are mature for each combination of region (1–5) and year between 1991 and 2015 as:

$$L_{50}=-a/b$$ Eq 2

Confidence intervals (2.5% and 97.5%) for estimates of size at maturity were then derived via bootstrapping procedures within SizeMat (N iterations = 1000).

We analysed trends in size at maturity of Southern Rock Lobster since 1991 for lobsters caught at the 20–30m depth contour as well as for lobsters caught from all depth contours. For each region (1–5), we then tested the null hypothesis of no temporal change in size at maturity (i.e. slope = 0) occurring since 1991 using linear regression with ANOVA in OriginPro® (p<0.05), with year as the dependent variable.

Catch per unit effort (CPUE) of legal-size Southern Rock Lobster is an indicator of relative abundance and was estimated from commercial catch returns that recorded the total catch (kg) landed and number of potlifts used by fishers each month between 1991 and 2015. Catch per unit effort in regions 1 to 5 in each year was measured as:

$${\mathrm{CPUE}}_{y,r}=\frac{\sum C_{y}{}_{,r}}{\sum f_{y,r}}$$ Eq 3

where CPUE_*y*,*r*_ is catch per unit effort estimated from the total weight of lobster catch (*C*)(kg) and total effort (*f*)(potlifts) in each year *y* within each region *r*.

We analysed trends in CPUE of legal-size Southern Rock Lobster since 1991 for lobsters caught at the 20–30m depth contour as well as for lobsters caught from all depth contours. For each region (1–5), significant trends in the CPUE of legal-size lobsters since 1991 were also tested for using linear regression with ANOVA in OriginPro^®^ (p<0.05), with year as the dependent variable.

Available sea-surface temperature data were collated for each region (1–5) between 1992 and 2015 from a one month average of all the highest available quality values that overlapped with cells of 0.02 degree x 0.02 degree resolution over the study area bounding 128.64°E to 141.09°E, 30.96°S to 38.61°S. All sea-surface temperature data were weighted by the area of overlap [49]. Two approaches were used to assess temporal and spatial trends in sea-surface temperature in regions 1 to 5 between 1992 and 2015. Firstly, we estimated annual averages of sea-surface temperature recorded for all depth contours, and at the 20–30m depth contour, in each region from 1992 and 2015, and tested for significance of increase or decrease of sea-surface temperature in each region over time using linear regression and ANOVA in OriginPro® (p<0.05), with year as the dependent variable. Secondly, for each region, we estimated annual sea-surface temperature anomalies at the 20-30m depth contour by subtracting the mean annual sea-surface temperature from the mean sea-surface temperature calculated over the study period (1992–2015). Annual sea-surface temperature anomalies were estimated for each year and region combination between 1992 and 2015, and contour maps for all regions at all depth contours were generated for two years with low and high sea-surface temperature anomalies (1994 and 2012, respectively).

Mean daily bottom temperature was calculated in region 3 and region 5 for all bottom data available from October to May between 1998 and 2015. Weather constraints resulted in bottom temperature data being unavailable for some months in the two regions where loggers were located. To enable analyses of inter-annual trends in bottom temperature, data were restricted to the summer months of December, January and February. In region 3, data were available from all summer months between 2008 and 2017. In region 5, available data were aggregated for all summer months sampled at Southend between 1998–2002, 2006–2012 and 2014–2015; Port McDonnell between 1998–1999, and 2002–2003; and from Robe between 1998–2001, 2006, 2009 and 2010. In each region, a significant increase or decrease in summer bottom temperatures over time was tested for using linear regression with ANOVA in OriginPro® (p<0.05), with year as the dependent variable. We also estimated summer-specific temperature anomalies for available data in region 3 between 2008 and 2016, and for region 5 between 1998 and 2015.

We used a first set of generalised linear models (GLM) [51] in SPSS (v24) to examine the effect of region, depth, sea-surface temperature, and relative abundance (CPUE) on estimates of size at maturity (L_50_) measured for all Southern Rock Lobster sampled between 1992 and 2015. Growth of Southern Rock Lobster to legal size in South Australia is reached in approximately four years [13] and we considered that growth during this time would incorporate the environmental conditions experienced by lobsters as they attained maturity. Consequently, for each region, we applied four-year average estimates of sea-surface temperature to yearly size at maturity (L_50_) estimates in the GLMs. Akaike’s information criterion, corrected for small sample sizes (AICc), was used to select the best models from a set of 35 candidate models developed *a priori*. AIC is useful as to compare candidate models derived from the same dataset and AICc provides for sample correction and greater penalty for each parameter. Models with smaller AICc values are preferred and models with changes in AICc (ΔAICc) greater than two have less support [52].

Examination of AICc values from the first set of GLMs indicated equal support (ΔAICc <2) from the two top models: 1) L_50_ ~ Region + Region*CPUE; and 2) L_50_ ~ Region*sea-surface temperature + Region*CPUE. Even though depth was not a significant predictor in these GLMs, we considered depth an important variable to account for because of strong evidence for its influence on size at maturity (L_50_), abundance (CPUE) and sea-surface temperature [18][50] (and see Results). Moreover, movement of fishing operations inshore over time (see Results) necessitated the need to reduce any potential confounding effect of depth on sea-surface temperature, CPUE, and size at maturity (L_50_) responses. Consequently, a second set of GLMs was developed, removing depth as a factor and using data restricted to lobsters sampled from depth contours between 20-30m. AICc was again used to select a final best model from a set of 32 candidate models developed *a priori*.

Collinearity diagnostics (Variance Inflation Factors [VIF]) obtained through regression analysis in SPSS (v24) were used to examine the presence of co-linearity between and among predictor variables in GLMs. Collinearity between all variables was negligible (all VIF less than three). Significance of best-fit model terms were tested using Wald Chi-square statistics and the percent change in deviance between the final model and null model was calculated as a measure of the amount of variation explained by the best-fit model [52].

## Results

### Fishing depth

**Fig 2:**
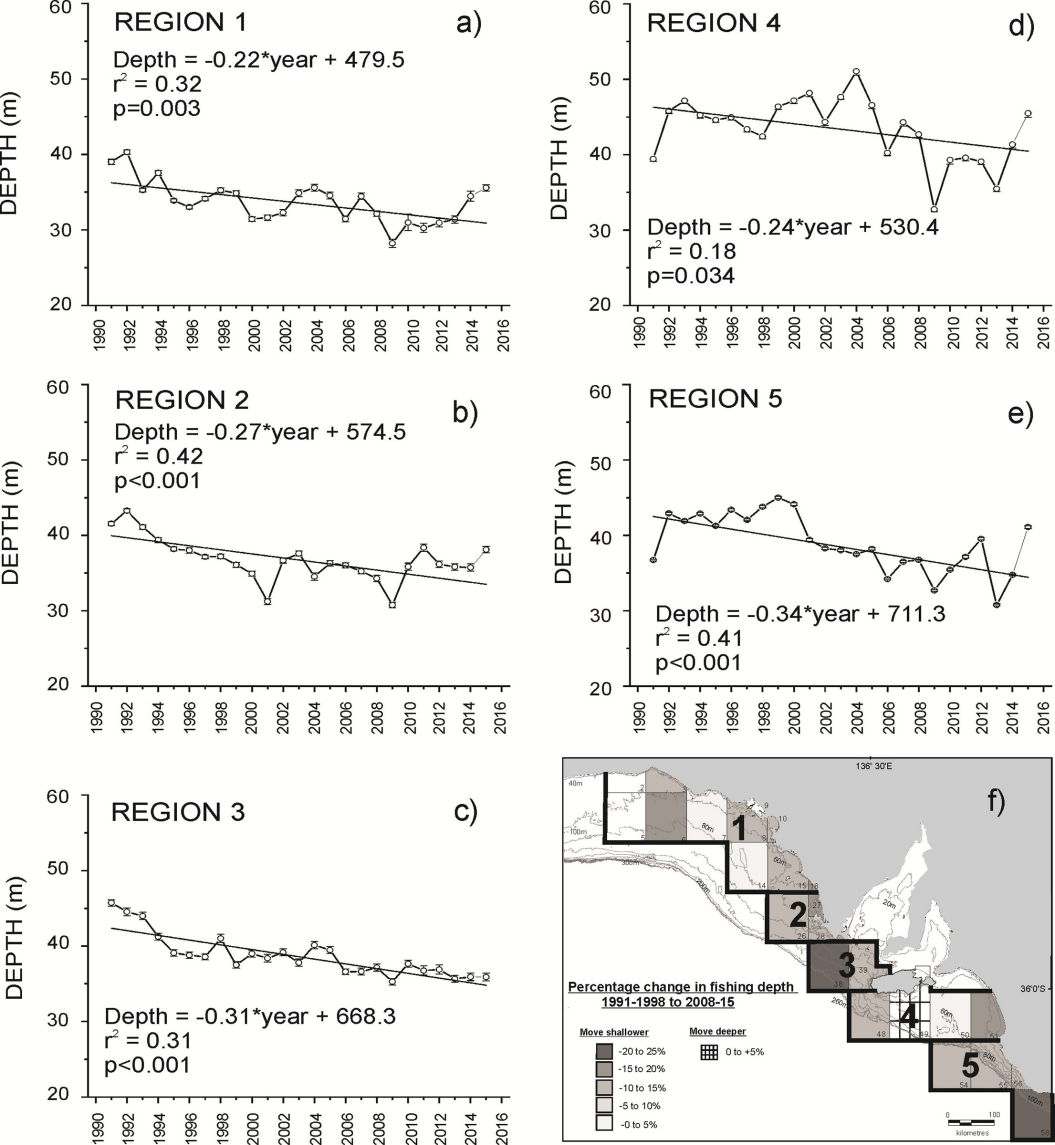
Temporal trends in fishing depth in regions 1–5 between 1991 and 2015 (graphs a-e) and percentage change in fishing depth within each Marine Fishing Area from 1991/98 to 2008/15 (f). Annual depth averages are presented ± standard error (SE). Negative % change equates to fishing operations moving shallower. Positive % change equates to fishing operations moving deeper.

Analyses of temporal trends in fishing depth indicated that fishing operations in all five regions have moved from deeper offshore areas to shallower inshore areas since 1991 (Fig 2A–2E). Between the period 1991–98 and 2008–15, decreases in the average depth of fishing operations were recorded in nearly all Marine Fishing Areas (Fig 2F). Changes in the depth of fishing operations of greater than 15% were recorded in Marine Fishing Areas 6 and 18 (region 1), Marine Fishing Area 27 (region 2), Marine Fishing Area 38 (region 3), Marine Fishing Area 51 (region 4) and Marine Fishing Area 58 (region 5) (Fig 2F). Only in Marine Fishing Area 49, off southern Kangaroo Island (region 4), was an increase (1%) in the depth of fishing recorded (Fig 2F).

### Size at maturity

**Fig 3:**
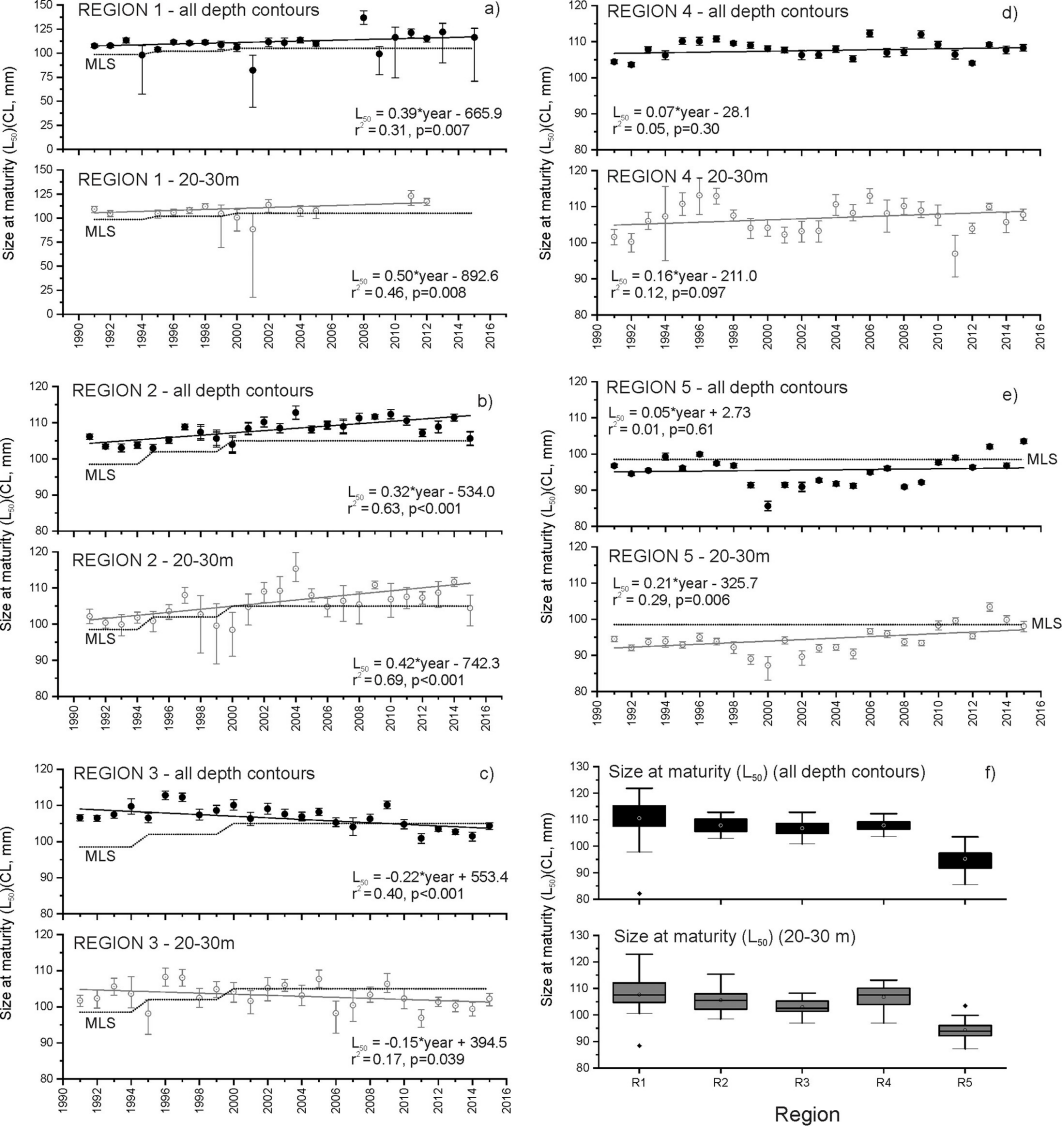
Temporal trends in the size at maturity of female Southern Rock Lobster caught from all depth contours and at the 20-30m depth contour in regions 1–5 (a-e, respectively) between 1991 and 2015. Annual averages are presented ± 95% CI. Minimum Legal Size (MLS) limits applicable to each region are shown by dotted line. Region 4 does not show MLS as it incorporates both the Northern Zone and Southern Zone management units, with different MLS limits. Box and whisker plot (f) showing variations in size at maturity (L_50_) from all depth contours and at the 20-30m depth contour in regions 1–5. Confidence Limits: box limits = 25th and 75th percentiles, whisker limits = 5th and 95th percentiles.

Significant increases in size at maturity were recorded for Southern Rock Lobster sampled from all depth contours in regions 1 and 2 since 1991 (ANOVA: region 1, F = 9.0, p = 0.007; region 2, F = 38.7, p<0.001) (Fig 3A and 3B). The highest rate of increase in size at maturity occurred in region 1 at 0.39±0.13 mm CL per year. In region 2, size at maturity increased at a rate of 0.32±0.06 mm CL per year. No significant changes in size at maturity were measured from lobsters sampled from all depth contours in regions 4 or 5 since 1991 (ANOVA: region 4, F = 1.13, p = 0.030; region 5, F = 0.26, p = 0.61). In contrast, size at maturity decreased significantly in region 3 at a rate of 0.22 ±0.06 mm per year (ANOVA: F = 15.4, p<0.001) (Fig 3C).

The temporal trends in size at maturity of lobsters caught at the 20–30m depth contour resembled those measured from all depth contours in each region (Fig 3A–3E). Since 1991, significant increases in size at maturity were recorded from Southern Rock Lobster caught at the 20–30m depth contour in regions 1, 2 and 5 (ANOVA: region 1, F = 10.23, p = 0.008; region 2, F = 51.84, p<0.001; region 5, F = 9.21, p = 0.006) (Fig 3A, 3B and 3E). Of these regions, the highest rate of increase in size at maturity also occurred in region 1 at 0.50±0.16 mm CL per year. In region 2, size at maturity increased at a rate of 0.42±0.06 mm CL per year and in region 5, the rate of increase in size at maturity was relatively slower at 0.21±0.07 mm CL per year (Fig 3A and 3E). No significant trend in size at maturity was measured in region 4 since 1991 (ANOVA: F = 3.00, p = 0.097). In region 3, similar to the trends in size at maturity of Southern Rock Lobster measured at all depth contours, size at maturity decreased significantly at a rate of 0.15 ±0.07 mm per year (ANOVA: F = 4.78, p = 0.039) (Fig 3C).

Regions 1, 2 and 3 comprise a large proportion of the Northern Zone management area, which has been the subject of two adjustments in the minimum legal size limit over the period of our study (1995: 98.5 to 102 mm CL; 2000: 102 to 105 mm CL). The increasing temporal trend in size at maturity in region 1 is highlighted in that over 70% of all annual estimates of size at maturity measured from all depth contours and at the 20–30m depth contour since 2000, are higher than the current minimum legal size limit of 105 mm (Fig 3A). Similarly in region 2, over 75% of all estimates of size at maturity since 2000 are higher than the current minimum legal size limit (Fig 3B). In contrast, the steady decrease in size at maturity recorded in region 3 since 1991 results in all estimates of size at maturity since 2010 being 0.2–7.7% lower than the current minimum legal size limit (Fig 3C).

Region 5 comprises a large proportion of the Southern Zone management area, where the minimum legal size limit is lower than in the Northern Zone, and has remained constant over the period of our study (98.5 mm CL). Since 1991, over 80% of all estimates of size at maturity measured from lobsters sampled at all depth contours and at the 20–30m depth contour have been lower than the minimum legal size limit (Fig 3D). However, the increases in size at maturity recorded in this region since 1991 now result in all estimates of size at maturity in the last 10 years being within ± 7.7% of the minimum legal size limit, with size at maturity estimates since 2010 among the highest on record.

Comparison of estimates of size maturity between regions indicate pronounced north to south spatial variation (Fig 3F). For lobsters sampled at all depths in region 1, size at maturity averaged 110.5±2.2 mm CL (range: 82.1–136.7 mm CL) between 1991 and 2015 (Fig 3F). In contrast, size at maturity of lobsters sampled at all depths in region 5 averaged 95.2±0.8 mm CL (range: 85.6–103.5 mm CL) over the same period (Fig 3F). Spatial trends in size at maturity were similar for lobsters sampled at the 20–30m depth contour, averaging 107.7±2.1 mm CL (range: 88.4–122.9 mm CL) between 1991 and 2015 (Fig 3F) in region 1, and 94.3±0.7 mm CL (range: 87.2–103.4 mm CL) in region 5 over the same period (Fig 3F).

### Relative abundance (Catch per unit effort)

**Fig 4:**
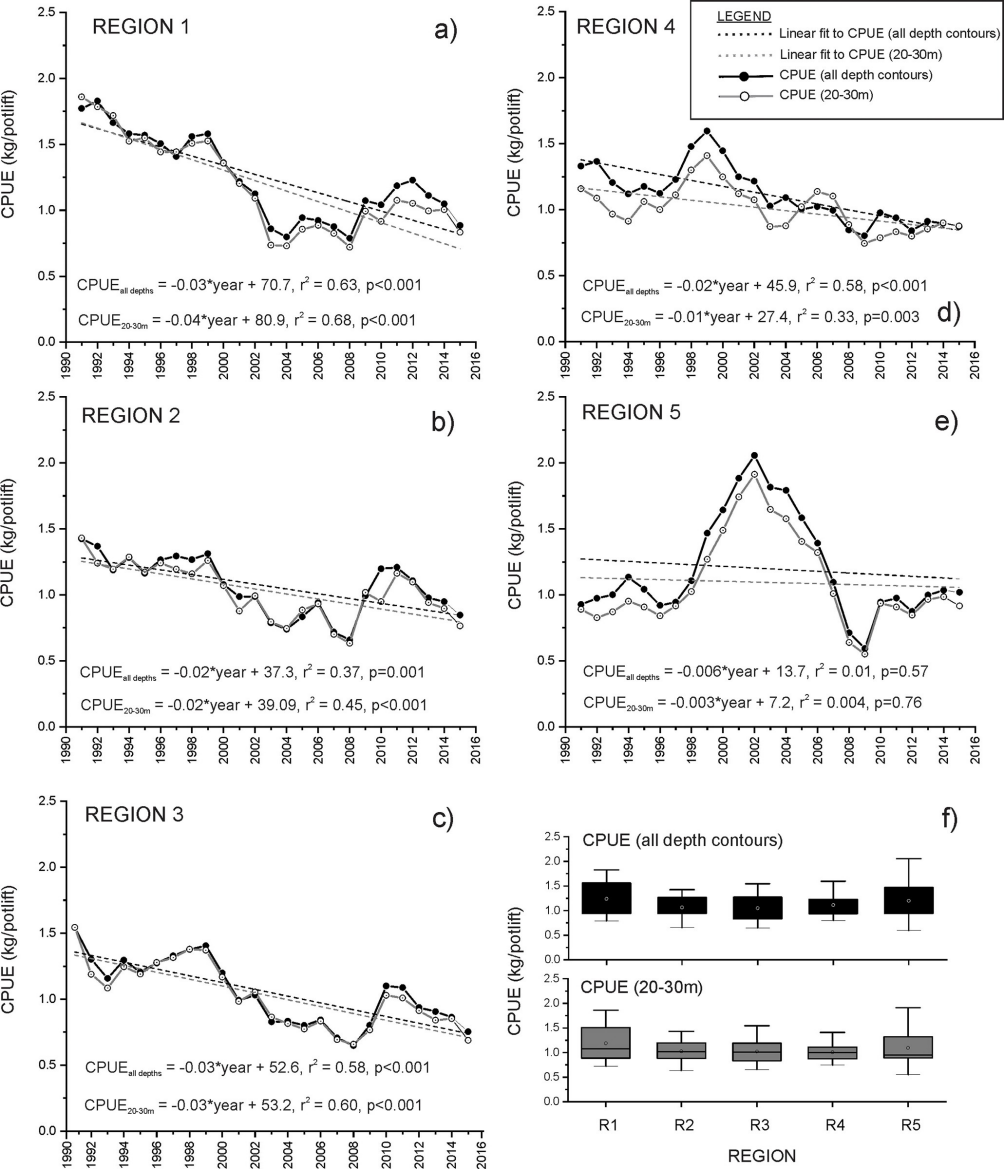
Temporal trends in the relative abundance (catch per unit effort, CPUE) of Southern Rock Lobster in regions 1–5 (a-e, respectively) caught at the 20–30m depth contour and at all depth contours between 1991 and 2015. Box and whisker plot (f) showing variations in CPUE from all depth contours and at the 20-30m depth contour in regions 1–5. Confidence Limits: box limits = 25th and 75th percentiles, whisker limits = 5th and 95th percentiles.

Catch per unit effort (CPUE), a measure of the relative abundance of legal-size Southern Rock Lobster, generally declined across the fishery since 1991 (Fig 4). Since 1991, CPUE measured from all depth contours targeted by fishing operations declined significantly in regions 1–4 (ANOVA results: F = 13.5–38.8, p≤0.001) (Fig 4A–4D) at a rate ranging between 0.02±0.004 kg/potlift per year (region 4)(Fig 4D) to 0.04±0.006 kg/potlift per year (region 1) (Fig 4A). In region 5, no significant trend in CPUE was evident likely due to a period of historically high levels of CPUE recorded between 1999 and 2006 (ANOVA: F = 0.33, p = 0.57) (Fig 4E).

Temporal trends in CPUE of Southern Rock Lobster caught at the 20–30m depth contour resembled those measured from all depth contours in each region (Fig 4). Significant declines in CPUE were recorded at 20–30m in regions 1–4 (ANOVA: regions 1–4, F = 11.1–49.3, all p ≤0.05). In all regions, annual estimates of CPUE recorded from relatively shallow inshore areas of 20–30m depth were slightly lower than estimates of CPUE measured from all depth contours. Differences were most pronounced in regions 1, 4 and 5, where annual estimates of CPUE measured at the 20–30m depth contour were an average of 5.1 (region 1) to 8.6% (region 4) less than annual estimates of CPUE measured from all depth contours fished in each region (Fig 4).

### Sea-surface temperature

**Fig 5:**
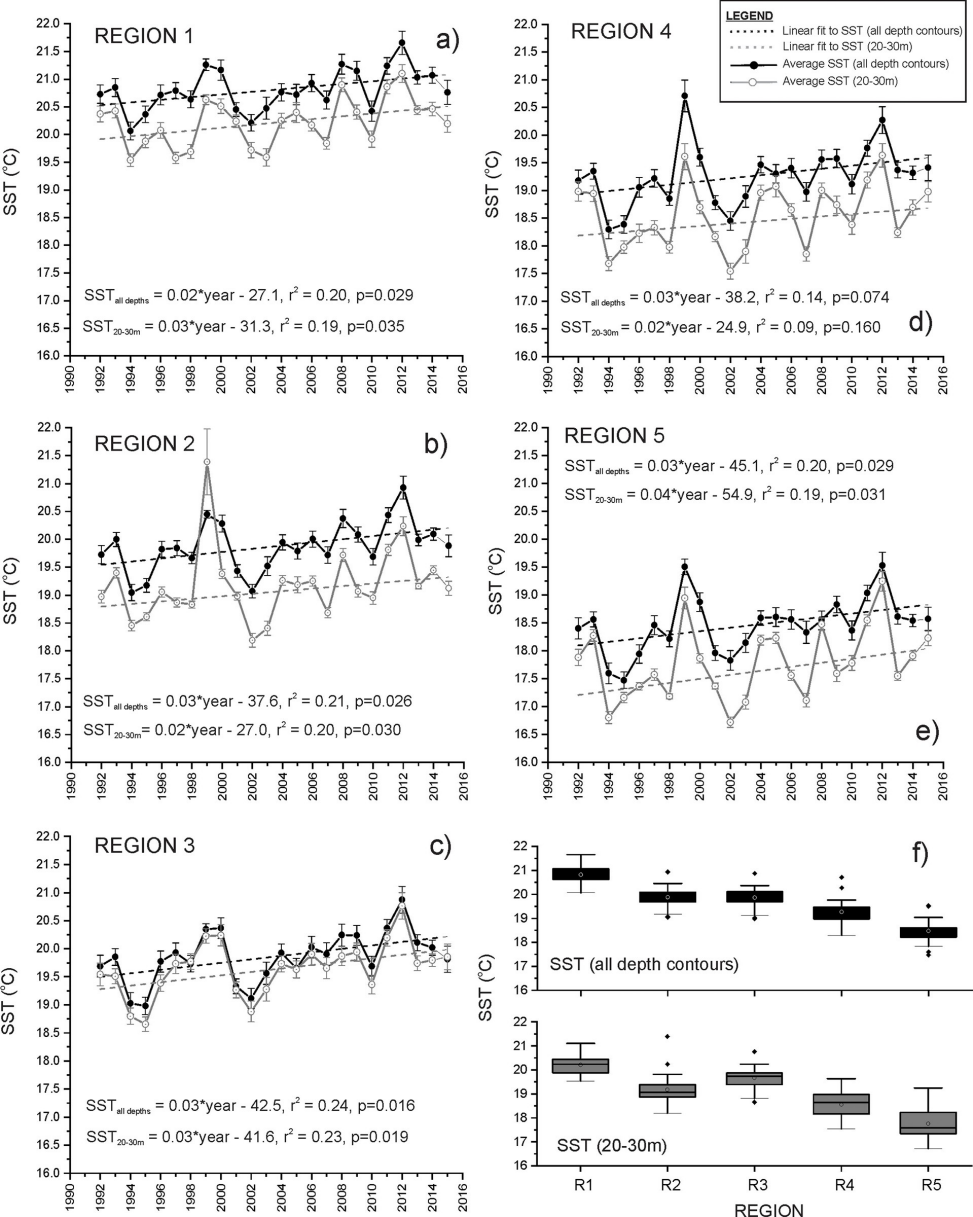
Temporal trends in sea-surface temperature in regions 1–5 (a-e, respectively) at the 20-30m depth contour and for all depth contours over the study area between 1992 and 2015. Annual averages of sea-surface temperature are presented ± standard error (SE). Box and whisker plot (f) showing variations in sea-surface temperature measured from all depth contours and at the 20-30m depth contour in regions 1–5. Confidence Limits: box limits = 25th and 75th percentiles, whisker limits = 5th and 95th percentiles.

The north to south spatial variation in estimates of mean sea-surface temperature in each region between 1992 and 2015 reflect the changes in latitude across South Australian shelf waters (Fig 5). In region 1 (31.5–34.0°S), mean estimates of sea-surface temperature measured from the all depth contours and at the 20–30m depth contour over this period were 20.8 ± 0.08°C (range: 20.1–21.7°C) and 20.2 ± 0.09°C (range: 19.5–21.1°C), respectively (Fig 5A and 5F). In contrast, in region 5 (37.0–39.0°S), in south-eastern South Australia, the mean estimate of sea-surface temperature between 1992 and 2015 from the all depth contours and at the 20–30m depth contour were 18.5 ± 0.10°C (range: 17.5–19.5°C) and 17.7 ± 0.13°C (range: 16.7–19.2°C), respectively (Fig 5E and 5F).

Estimates of mean annual sea-surface temperature measured from all depth contours generally increased in each region across South Australia since 1992 (Fig 5A–5E). Regression analyses indicated significant increases in average sea-surface temperature in regions 1, 2, 3, and 5 since 1992 (ANOVA results, F = 5.48–6.84, all p≤0.05) (Fig 5A, 5B, 5C and 5E). Average sea-surface temperature increased in these four regions at a rate ranging between 0.02 ±0.01 ^o^C.year^-1^ in region 1 (Fig 5A) to 0.03 ±0.01 ^o^C.year^-1^ in region 5 (Fig 5E). No significant trend in sea-surface temperature was observed in region 4 between 1991 and 2015 (ANOVA, F = 3.53, p = 0.074) (Fig 5D). Similar increasing trends in average sea-surface temperature were measured at the 20–30m depth contour in regions 1, 2, 3, and 5 since 1992 (ANOVA, F = 5.03–6.46, all p≤0.05) (Fig 5A–5E), however average sea-surface temperatures in each region measured at 20–30m were relatively lower in all years when compared to average sea-surface temperature measured from all depth contours (Fig 5A–5E).

**Fig 6:**
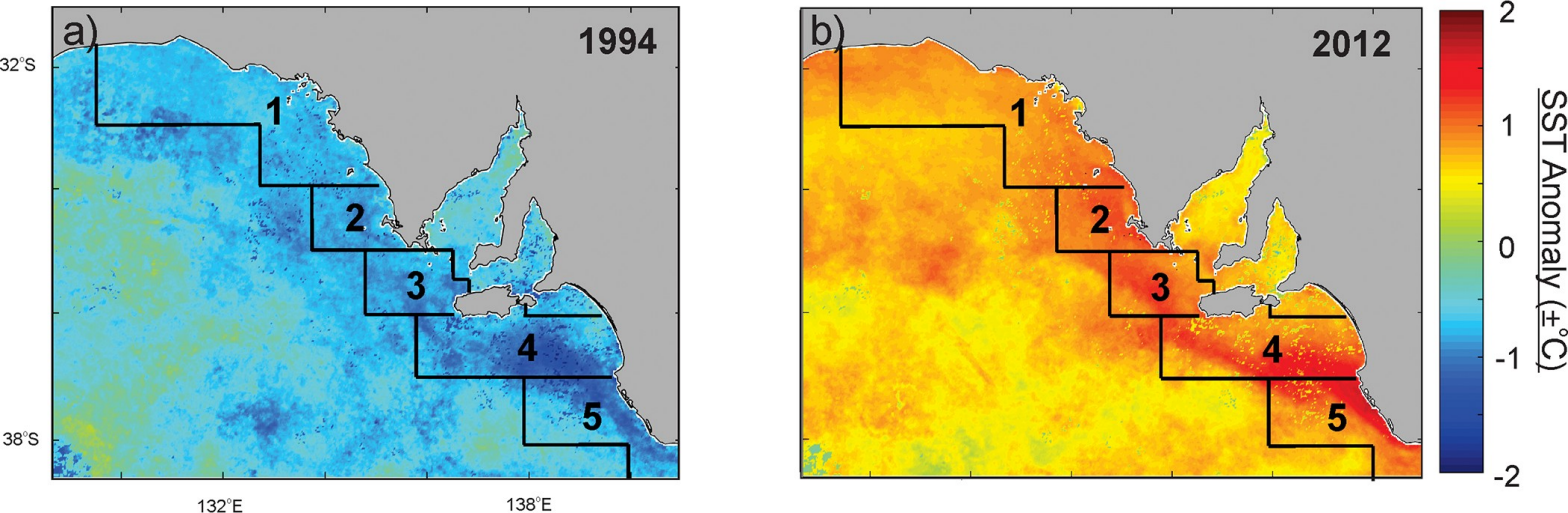
Sea-surface temperature anomalies calculated at the 20-30m depth contour for two years with low and high sea-surface temperature anomalies (1994 (a) and 2012 (b), respectively).

Annual anomalies in sea-surface temperature measured relative to the average sea-surface temperature recorded between 1992 and 2015 indicate that the increasing trends in sea-surface temperature in each region in South Australia are largely a result of higher than average sea-surface temperatures (positive anomalies) recorded in the last 10 years. Since 2006, positive sea-surface temperature anomalies have been recorded in each region in at least seven years. Mapping of sea-surface temperature anomalies in 2012 indicate sea-surface temperatures in each region 0.84–1.06°C above the 1992–2015 average with positive annual sea-surface temperature anomalies widespread across regions 3–5 (Fig 6B). In contrast, sea-surface temperature anomalies in 1994 were 0.75–0.97°C below the 1992–2015 average in each region with negative sea-surface temperature anomalies most pronounced in regions 4 and 5 (Fig 6A).

### Bottom temperature

**Fig 7:**
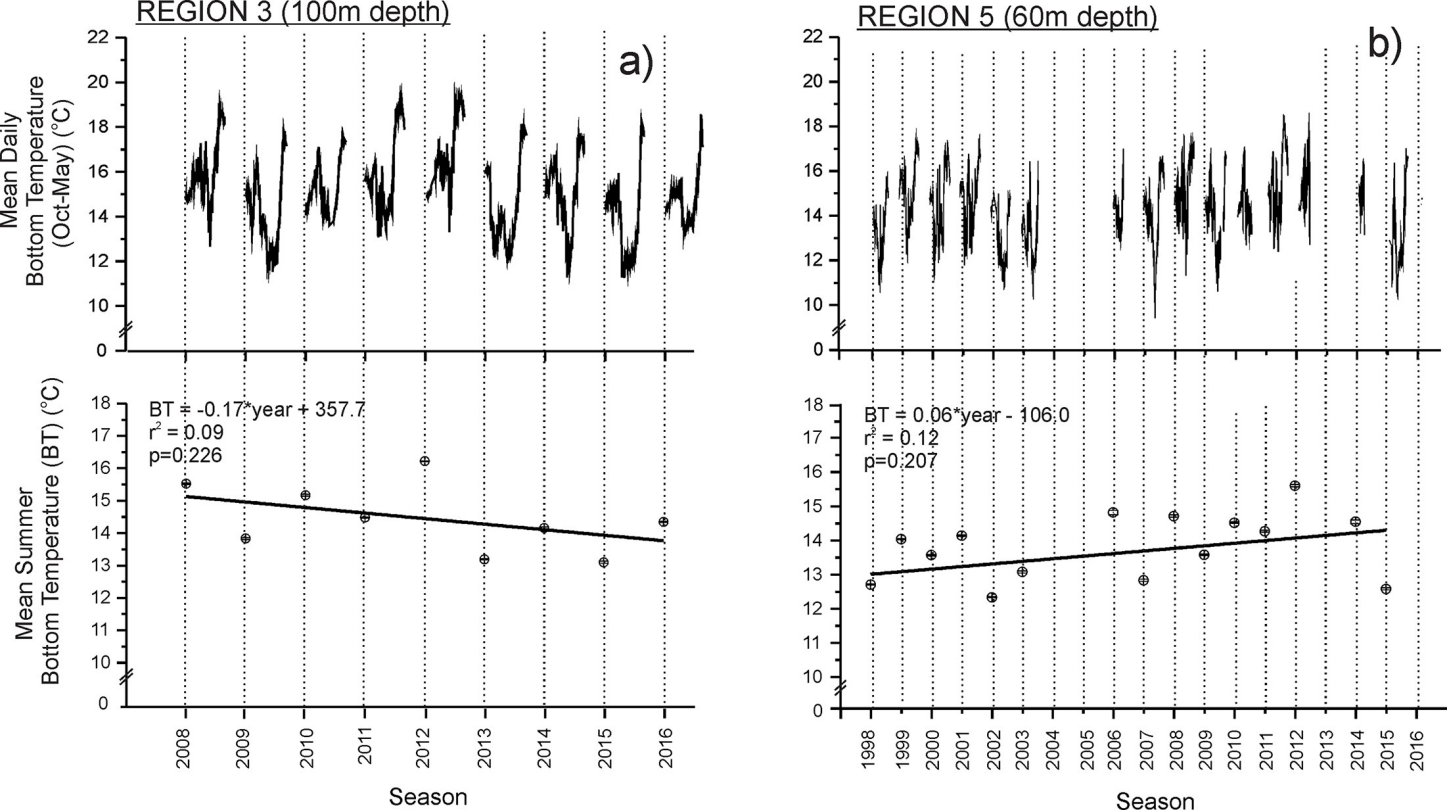
Temporal trends in bottom temperature measured from the IMOS mooring deployed at 100 m depth in region 3 between December 2008 and February 2017, and from onset® StowAway® TidbiT® bottom loggers deployed at 60 m depth in Region 5 between December 1998 and February 2016. Mean daily bottom temperature is presented for data available from October to May. Mean summer bottom temperature are presented for data averaged from December, January and February. Mean summer bottom temperatures are presented ± standard error (SE).

The temporal scales at which bottom temperature data were collected in region 3 (2008–2016) and region 5 (1998–2016) do not match that measured for mean sea-surface temperature (1992–2015), however, available bottom temperature data in these regions give an indication of the recent trends in temperature that may be influencing patterns of lobster growth and size at maturity. Regression analyses indicated a non-significant decrease in mean summer bottom temperature between 2008 and 2016 in region 3 (ANOVA: F = 1.76, p = 0.226) (Fig 7A). Mean summer estimates of bottom temperature in region 3 from 2013 to 2016 were between 0.10 and 1.34°C below the 2008–2016 average also indicating a decreasing trend in bottom temperature in this region. In region 5, regression analyses indicated a non-significant increase in mean summer bottom temperature between 1998 and 2015 (ANOVA: F = 1.76, p = 0.207) (Fig 7B). In contrast to region 3, six of the nine mean summer estimates of bottom temperature in region 5 from 2006 to 2015 were above the 1998–2015 summer average, indicative of an increasing trend in bottom temperature in this region.

### Size at maturity responses

Residual plots and AICc values indicated that the most suitable GLMs to model the effects of region, sea-surface temperature, CPUE and their interactions on size at maturity incorporated a Gamma error structure and log link function. The Gamma distribution captured the strong right positive skew of annual L_50_ estimates in each region. The top candidate sets of models including the ‘null’ model, are presented in Table 2. Values of AICc indicated that the most well supported model included region, CPUE and a region*sea-surface temperature interaction. This model explained 63.8% of the deviance from the null model (100*[0.458 – 0.166]/0.458) (Table 2). Wald Chi-square tests indicated that all model terms were significant (Wald Chi-square = 7.5–16.9, all p < 0.05) demonstrating that L_50_ is strongly region specific, and best explained by both CPUE and the interaction between region and sea-surface temperature. Examination of GLM parameter estimates and Wald Chi-square statistics for individual parameters of the most well supported model indicated a significant negative relationship between L_50_ and CPUE (β = -0.043, Standardised beta = -0.001, Wald Chi-square = 7.463, p = 0.006). Significant negative relationships between L_50_ and the region*sea-surface temperature interaction in Region 3 (β = -0.054, Standardised beta = -0.001, Wald Chi-square = 4.137, p = 0.042) and Region 4 (β = -0.062, Standardised beta = -0.001, Wald Chi-square = 4.952, p = 0.026) (Appendix 1) were also observed, indicating respective declines in size at maturity of -0.054 and -0.062 mm in these regions for every one degree increase in sea-surface temperature. Conversely, there was a significant positive relationship between L_50_ and the region*sea-surface temperature interaction in Region 5 (β = 0.049, Standardised beta = 0.001, Wald Chi-square = 3.894, p = 0.048) indicating an increase in size at maturity of 0.049 mm in region 5 for every one degree increase in sea-surface temperature (Appendix 1).

**Table 2:** Results of the top 12 Generalised Linear Models (GLM) showing the effect of region, sea-surface temperature (SST), and relative abundance (catch per unit effort (CPUE)) on changes in size at maturity (L_50_) of Southern Rock Lobster. Region was included as a categorical fixed effect. Dev: deviance; LL: log likelihood; AICc: Akaike’s information criterion corrected for small sample sizes; ΔAICc: change in AIC between the best and candidate model. The null (intercept only) and full (all factors of interest) models are also listed.

Model	k	Dev	LL	AICc	ΔAICc
L_50_ ~ Region + CPUE + Region*SST	5	0.166	-306.2	639.7	0.0
L_50_ ~ Region + CPUE	4	0.191	-313.9	642.8	3.2
L_50_ ~ Region + Region*SST	4	0.178	-309.8	644.3	4.7
L_50_ ~ Region + CPUE + SST	5	0.190	-313.6	644.6	5.0
L_50_ ~ Region + Region*CPUE	4	0.179	-310.3	645.3	5.6
L_50_ ~ Region + CPUE + SST+ Region*SST + Region*CPUE (full model)	7	0.159	-303.8	645.5	5.9
L_50_ ~ CPUE + Region*SST	4	0.192	-314.1	645.6	6.0
L_50_ ~ Region	3	0.203	-317.0	646.8	7.2
L_50_ ~ Region + SST+ Region*CPUE	5	0.179	-310.2	647.7	8.0
L_50_ ~ Region*SST + Region*CPUE	2	0.179	-310.4	648.1	8.4
L_50_ ~ Region + SST	4	0.203	-317.0	649.1	9.5
L_50_ ~ Region*SST	1	0.204	-317.5	650.1	10.5
L_50_ ~1 (null model)	1	0.458	-361.5	727.2	87.5

## Discussion

### Overview

Fisheries are subject to a variety of regulatory controls that are designed to manage the fraction of mature biomass that is harvested, thereby maintaining rates of egg production and larval settlement at a level where recruitment is not impaired. Where minimum legal size limits are applied, and informed by measures of size at maturity (L_50_), an understanding of how size at maturity varies both temporally and spatially is crucial to ensuring that management objectives are meeting their intent. There is a general paucity of information relating to long-term spatio-temporal responses in size at maturity in spiny lobster populations. Southern Rock Lobster are a good species to examine spatio-temporal size at maturity responses to habitat conditions, because they exhibit limited movement in their benthic life history phase [53][54] and are therefore likely to integrate the signals of their local environmental conditions throughout their life. Our study was fortuitous in having available a long time series of biological data collected from a Southern Rock Lobster stock assessment monitoring program originating in 1991, and highlights spatial heterogeneity in size at maturity of Southern Rock Lobster over an area spanning approximately 229,000 km^2^ as well as temporal differences in size at maturity occurring over 25 years.

### Spatio-temporal patterns in size at maturity

Higher annual estimates of size at maturity were recorded in the north-western areas of the fishery (region 1) relative to the south-east (region 5). This latitudinal pattern is similar to the north-south decline in estimates of size at maturity described for Southern Rock Lobster in Tasmania [32][55], as well as spatial patterns in size at maturity measured previously in South Australia [17][56]. The latitudinal pattern in estimates of size at maturity observed in our study is also spatially consistent with growth rate estimates of Southern Rock Lobster recorded over the same area [13], where relatively higher growth rates of mature females were identified in the north western and western parts of the fishery (regions 1 and 2 –this study) compared to south-eastern localities (region 5).

Linear regressions of size at maturity measured between 1991 and 2015 in regions 1, 2, 3 indicated significant departures from the null hypothesis of zero slope (trend) for lobsters caught at the 20–30m depth contour as well as for lobsters caught from all depth contours. Size at maturity of lobsters also increased significantly in region 5 for lobsters caught at the 20–30m depth contour. However, the nature (slope) of the temporal responses in size at maturity recorded in each region since 1991 differed. The increasing temporal trend in size at maturity in regions 1, 2 and 5, and historically high annual estimates of size at maturity calculated in these regions since 2000 indicate that the current minimum legal size limits in these regions may not be providing the same amount of protection to egg production and Southern Rock Lobster biomass compared to what they have historically. Conversely, the steady decreases in size at maturity in region 3 since 1991 indicates that the current minimum legal size limit in this region may be providing a higher degree of protection to egg production and biomass than it was in 2000. As indicated in egg-per-recruit modelling for female American lobster [57], egg production tends to increase as size at maturity declines because of a higher chance of spawning prior to landing, but this relationship is ultimately dependent on the intensity of harvesting (harvest fraction) and strength of size-based selection in the fishery. In light of the changes in size at maturity measured in our study throughout different parts of the South Australia Rock Lobster Fishery, spatially resolved egg-per recruit modelling that considers the temporal trends in size at maturity presented herein would be a logical next step for stock assessment research in South Australia and should be considered more broadly as a priority for national assessment that considers 20% unfished egg production as a limit reference point [21].

### What is driving patterns in size at maturity?

The low genetic diversity of Southern Rock Lobster across southern Australia is ascribed to a long planktonic phase and wide ranging larval dispersal, and points to the spatio-temporal differences in size at maturity recorded across the South Australian Rock Lobster Fishery as being phenotypic rather than genotypic [58][59]. Of the abiotic and biotic processes likely to influence growth and resulting size at maturity in lobster populations, climatic and density-dependent factors have received the most attention. However, attributing changes in size at maturity to either environmental and/or density-dependent factors is made difficult by the need to experimentally control for the influence of each factor. Neither temperature nor density could be attributed to decreasing temporal trends in size at maturity in female American Lobster (*Homarus americanus*) [60]. In Southern Rock Lobster, estimates of size at maturity in fished versus unfished areas in Tasmania were found to be similar despite large differences in lobster density [32], indicating that size at maturity is regulated by factors other than density. In contrast, a study of size at maturity in the Spiny Lobster (*Palinurus elephas*) recorded smaller estimates of size at maturity inside a marine reserve characterised by relatively high densities of lobsters, but also noted that the patterns in size at maturity observed may have been environmentally driven [61].

Our data did not allow comparison of size at maturity in fished versus unfished components of the population. We hypothesised that size at maturity was driven by either temperature or density (CPUE), and the results of our best fitting GLM indicate that neither hypothesis could be rejected, with our most well supported model including the significant factors of region, CPUE and a region*sea-surface temperature interaction. Standardised beta coefficients within our best fitting GLM also did not help explain which factor, CPUE or regional SST trends, most contribute to the variations in size at maturity observed. The declining trends in fishery CPUE across all five regions of the South Australian Rock Lobster Fishery since 1991 indicate a widespread decrease in the density of Southern Rock Lobster. Catch rate is regularly used in crustacean stock assessments [15][16] as an indicator of relative abundance and the trends in CPUE reported in our study are similar to those reported in other Southern Rock Lobster fisheries across south-eastern Australia [19]. The significant negative relationship between CPUE and size at maturity within our GLM is supportive of the hypothesis that a lower density of Southern Rock Lobster is increasing growth rates, resulting in increases in size at maturity, and could help explain the increases in size at maturity observed in regions 1, 2 and 5 since 1991 under declining trends in relative abundance. However, the significant declining trends in size at maturity in region 3 since 1991 and lack of temporal responses in size at maturity in region 4 under similar declines in relative abundance, are at odds with the hypothesis that size at maturity is being shaped by density-dependent processes.

The declining gradient in size at maturity estimates from north to south recorded in our study support the hypothesis that water temperature is a key factor determining growth rates and resulting size at maturity in Southern Rock Lobster populations. The observed increases in size at maturity since 1991 in regions 1, 2 and 5 are also consistent with the increasing sea-surface temperature measured in these regions. However, the decreases in size at maturity observed in region 3, near western Kangaroo Island (Fig 1), are at odds with the increases in sea-surface temperature recorded in this region. The depth to which satellite derived sea-surface temperature measurements represent sub-surface temperatures in the benthic environment is likely to be dependent on local oceanographic conditions and is acknowledged as not being spatially or temporally consistent [36]. Comparison of data relating to sea-surface and bottom temperatures derived from bottom loggers in our study is made difficult by the temporal and spatial mismatches over which the data has been collected in each region. An understanding of the physical oceanographic characteristics occurring in each region gained from previous studies, coupled with information relating to the annual sea-surface and bottom temperature data presented, provides insight into the processes that may be shaping the patterns of size at maturity observed.

The physical oceanographic characteristics of shelf waters between the eastern Great Australian Bight and the Bonney Coast in south-eastern South Australia are only recently documented [37][38][40]. The continental shelf of the eastern Great Australian Bight is broad and relatively shallow resulting in summer warming of shelf waters [42], vertical mixing and formation of the Great Australian Bight warm pool [40] in regions 1 and 2. The presence of this vertically well-mixed warm pool of water [40] in regions 1 and 2 indicates that the increasing trends in sea-surface temperature recorded since 1991 may be good proxies for the trends in bottom temperature in these regions, and be driving the increases in size at maturity observed.

In contrast, the relatively narrow shelf and steep bathymetry of southern Eyre Peninsula and south-western Kangaroo Island (region 3), and the Bonney Coast (region 5) support two to four near-shore upwelling events per summer [37][41]. The variable strength and intensity of upwelling in these regions makes the potential relationship between sea-surface and bottom temperature less clear. For example, in region 3, the positive trend in sea-surface temperature measured since 1991 contrasts with the negative trend in bottom temperature collected from 100m since 2008. In this region, upwelled water is drawn onto shelf waters shallower than 100m during summer, with evidence of the existence of a bottom cold pool occurring below a warm surface layer near the mouth of Spencer Gulf, <10 km from the coast in waters of approximately 30m depth [40].

Although bottom temperature data recorded in our study from region 3 are only available since 2008, observations indicate that between 2013 and 2016, a total of 43 days recorded bottom temperatures of <12°C. This contrasts with bottom temperature data recorded between 2008 and 2012, when only 5 days recorded bottom temperatures of <12°C. These observations indicate the existence of temporal variations in the frequency and intensity of upwelling in this region with a decreasing trend in bottom temperature apparent since 2008, and negative bottom temperature anomalies observed between 2013 and 2016. Consequently, there is evidence that upwelling has been more intense in region 3 since 2013, and is acting to lower bottom temperatures, and decrease growth rates and size at maturity of lobsters in this region. Longer time series of bottom temperature data are required to draw better conclusions as to the nature of trends in upwelling frequency and intensity in region 3, and any resulting effects on size at maturity of Southern Rock Lobster, however it is worth noting that upwelling events are predicted to intensify under global climate warming [62]. If so, it is possible that estimates of size at maturity of Southern Rock Lobster in this region could decrease further.

**Fig 8:**
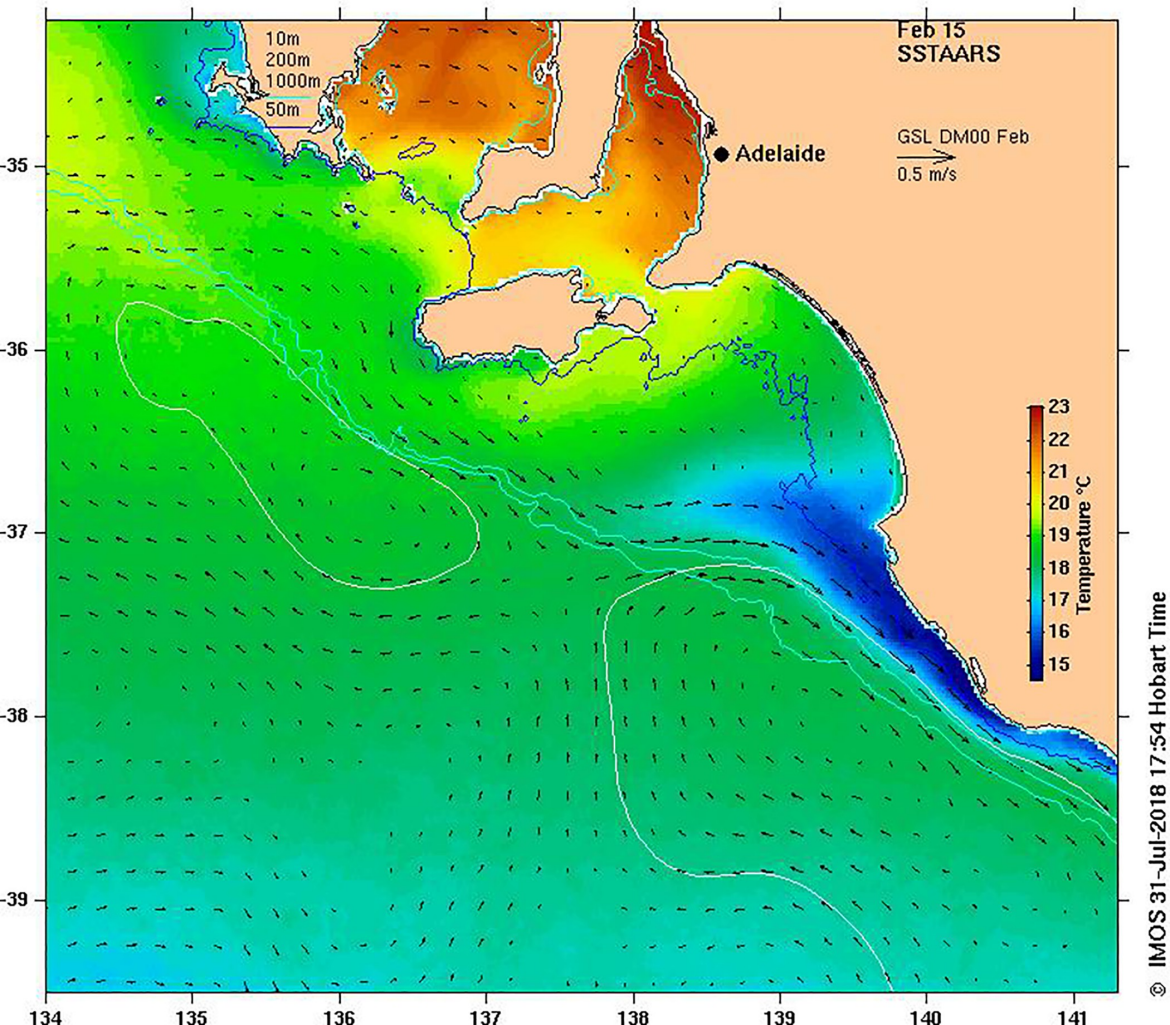
Example of typical summertime sea-surface temperature in south-eastern South Australia. The image shows the 25 year climatological condition for February from the Sea-surface Temperature Atlas of Australian Regional Seas (SSTAARS) provided by the Bureau of Meteorology and available through the Integrated Marine Observing System Ocean Current website (oceancurrent.imos.org.au/product). Areas of dark blue (<16°C) indicate cold water upwelling events.

No significant change in size at maturity or sea-surface temperature was detected in region 4 since 1991. Region 4 is also influenced by upwelling events that originate to the south east in region 5, where along-shore wind stress from predominating south-easterly winds in summer and a narrow shelf leads to upwelling of water from depths of 250m that may be transported up to 400km over a 10 day period [63][38]. The intrusion of upwelled water onto the shelf in these regions is shown in Fig 8. No bottom temperature measurements are available from region 4 so the nature of the relationship between upwelling, sea-surface temperature, bottom temperature and size at maturity remains largely unknown. In region 5, corresponding increases in size at maturity and sea-surface temperature since 1991, and bottom temperature since 1998, support temperature as a key factor determining size at maturity in this region. Similar to region 3, region 5 is also influenced by upwelling. However, unlike region 3, upwelling more regularly reaches the surface in region 5 [37], resulting in a well-mixed water column across the shelf, and as a consequence, trends in sea-surface temperature in this region are likely to be a good proxy for trends in bottom temperature. It should also be noted that bottom temperature data derived from loggers in region 5 have previously been shown to be strongly correlated with a semi-global ocean circulation model [64] indicating that data recorded from loggers are representative of actual bottom temperatures across the region.

### Potential biases

The presence of ovigerous setae on female spiny lobsters is often used as a cost-effective diagnostic tool for assessing size at maturity in stock assessment, but has been criticised for having potential bias towards larger estimates of size at maturity if setae are not retained during the moult phase [25][65]. Research using tag-recapture data [65] detected no loss of ovigerous setae in Southern Rock Lobster following moulting. However, the study did identify a seasonal (within year) component to size at maturity estimates, which the authors attributed to factors that may affect catch composition (e.g. Southern Rock Lobster movement, catchability), and recommended that sampling occur outside major biological events of moulting, mating and larval release. We consider that there is limited potential for this bias to exist in our study because our analyses was restricted to data collected during the fishing season between October and May, and therefore outside the breeding and moulting period for female Southern Rock Lobster in South Australia [15][16].

The limitations associated with the use of fishery-dependent data should also be noted. For example, catch composition can be affected by changes in fishing behaviour. Previous research in the South Australian Rock Lobster Fishery, identified a negative relationship between estimates of growth, size at maturity and depth [13][18] so our study assessed the need to account for changes in the depth of fishing over time. The recorded movement of fishing operations to shallower inshore fishing grounds across all areas of the fishery since 1991 can be attributed to changes in market demand, where inshore lobsters that are ‘red’ in colour and of relatively higher value became favoured over ‘speckled’ or ‘white’ lobsters typically found in offshore grounds [66][50]. Our study accounted for any biases in size at maturity estimates associated with these changes in fishing behaviour by restricting analyses to Southern Rock Lobster caught at 20–30m depth. The influence of fishing gear type on the size of lobsters sampled should also be acknowledged. Within South Australia, lobsters <70 and >210 mm CL are rarely landed by commercial fishing pots, which is consistent with the size selectivity of trap-caught spiny lobsters in other fisheries [67]. As a result, the data used to estimate size of maturity were limited to these specific size classes.

That withstanding, it is important to consider the overall limitations of using fishery-dependent CPUE as a measure of abundance in our study, as catch rates can also be influenced by factors such as gear selectivity, changes in fishing patterns, fleet efficiency or fleet dynamics over time [68]. Within the South Australian Southern Rock lobster Fishery, two lines of evidence suggest that catch rate trends largely reflect overall lobster abundance. Firstly, trends are highly consistent across large spatial scales. For example, across regions 1 to 3 of this study, catch rate simultaneously decreased from 1991 to 2008, increased briefly to 2011, before again decreasing over the next four seasons. In addition, catch rate trends are shown in this study and others [50] to be are consistent with depth in South Australia. Overall, these observations suggest recruitment in the fishery occurs across large spatial scales with any changes in CPUE sufficiently captured in the high level of seasonal and spatial coverage (>1 million potlifts annually) used in catch rate estimation.

Secondly, previous analyses [15][16] have highlighted that when nominal catch rates are standardised for factors such as year, month, depth, MFA, mean weight, licence and consumer price index (CPI), both nominal and standardised time series are closely aligned. However, it is important to highlight that while no difference was observed between the approaches, the standardisation did not include some factors known to have important effects on lobster catch rate in other lobster fisheries within south-eastern Australia. Specifically, data pertaining to the effects of ‘vessel’ were not available as part of the South Australian analyses. This component has been identified as a specific factor known to impact on lobster catch rates within the Southern Rock Lobster fishery of Victoria, Australia [69].

### Environmental and management implications

Marine climate research in Australia indicates that ocean temperatures have increased in the last century and that rates of warming have risen each decade. Average sea-surface temperature around Australia measured between 1992 and 2011 was 0.68°C higher than measured for period 1910–1929, and rates of warming also rose from 0.08°C/decade in 1920–2011 to 0.11°C/decade in 1950–2011 [70]. Further warming is predicted, with sea-surface temperatures expected to rise 2–3°C in Australia by 2070 [70]. The 0.02–0.03°C per year increases in sea-surface temperature recorded in our study since 1992 are the first documented for the region and are nearly double that reported for the Australian region on average, highlighting the importance of examining biophysical relationships at spatially relevant scales.

The impacts of ocean warming via climate change are now manifested in changes in the distribution, abundance and reproductive phenology of fish stocks [71][72][73][74][75], structure of marine communities [76][77][78][79] and trophic interactions [80][81]. In southern Australia, the impacts of climate change on key marine species have been predicted through risk assessments [82] and modelling approaches [23][83]. Observed impacts of climate change to fisheries remain largely unquantified, however increases in the growth rates of Western Rock Lobster have been linked to progressive ocean warming in Western Australia [2][30]. The relatively high rates of increase of sea-surface temperature and size at maturity of Southern Rock Lobster recorded in our study from the well mixed waters of regions 1 and 2 since 1991, indicate that the growth rates of lobsters in these regions may also be increasing in response to ocean warming associated with global climate change. Changes in size at maturity and bottom temperature in region 3 since 2008 and region 5 since 1998 also indicate that the growth rates of Southern Rock Lobster are responding to variations in the oceanographic processes in these regions.

How current harvest dynamics, management rules, and changes in size at maturity interact to influence Southern Rock Lobster populations is a key question for fisheries management. Any excessive removal of immature Southern Rock Lobster has the potential to lead to less egg production, less yield per recruit, and growth overfishing [84]. Considering the broad scale declines in recruitment already recorded across southern Australia [15][16][19][85] and that current minimum legal size limits in regions 1, 2 and 5 will not provide the same level of protection to egg production and biomass of Southern Rock Lobster populations in the future, it is important that management responses are able to adapt quickly to any biological changes observed. Future management would benefit from research that assesses the sensitivity of Southern Rock Lobster populations and fishery economic yield to the changes in size at maturity predicted in our study under varying minimum legal size limits and harvest rates. Ongoing collection of spatially relevant growth and size at maturity data is also recommended to refine the inputs of stock assessment models and inform future management strategies in the future. As ocean temperatures are predicted to warm further, our study highlights the need for the collection of better oceanographic information for integration into stock assessment models. Collection of bottom temperature data from spatially representative sites for incorporation into oceanographic models is seen as a priority and would enhance the understanding of environmental processes affecting a range of benthic fisheries. Such information could be integrated into future stock assessment models to enhance harvest strategy development, allow timely adaptive management decisions and increase the resilience of fisheries to the impacts of climate change.

### Appendices

**Appendix 1:** Parameter estimates of top generalised linear model (L_50_ ~ Region + CPUE + Region*SST).

Parameter	β	Standardised beta	SE	Wald Chi-Square	df	Sig.
(Intercept)	3.719		0.45	69.307	1	**0.000**
[Region = 1]	-0.446	-0.235	0.97	0.210	1	0.647
[Region = 2]	1.247	0.594	0.65	3.695	1	0.055
[Region = 3]	2.009	0.987	0.67	9.027	1	**0.003**
[Region = 4]	2.139	1.058	0.67	10.076	1	**0.002**
[Region = 5]	0a					
CPUE	-0.043	-0.001	0.02	7.463	1	**0.006**
[Region = 1] * SST	0.073	0.002	0.04	2.802	1	0.094
[Region = 2] * SST	-0.014	0.000	0.03	0.305	1	0.581
[Region = 3] * SST	-0.054	-0.001	0.03	4.137	1	**0.042**
[Region = 4] * SST	-0.062	-0.001	0.03	4.952	1	**0.026**
[Region = 5] * SST	0.049	0.001	0.03	3.894	1	**0.048**
